# Damage Identification for Large Span Structure Based on Multiscale Inputs to Artificial Neural Networks

**DOI:** 10.1155/2014/540806

**Published:** 2014-05-25

**Authors:** Wei Lu, Jun Teng, Yan Cui

**Affiliations:** Shenzhen Graduate School, Harbin Institute of Technology, Shenzhen 518055, China

## Abstract

In structural health monitoring system, little research on the damage identification from different types of sensors applied to large span structure has been done in the field. In fact, it is significant to estimate the whole structural safety if the multitype sensors or multiscale measurements are used in application of structural health monitoring and the damage identification for large span structure. A methodology to combine the local and global measurements in noisy environments based on artificial neural network is proposed in this paper. For a real large span structure, the capacity of the methodology is validated, including the decision on damage placement, the discussions on the number of the sensors, and the optimal parameters for artificial neural networks. Furthermore, the noisy environments in different levels are simulated to demonstrate the robustness and effectiveness of the proposed approach.

## 1. Introduction

Structural damage identification has taken increasing attention from the scientific and engineering communities because the unpredicted structural failure could cause catastrophic, economic, and human life loss. A reliable and effective damage identification methodology is significant to maintain safety and capacity of structures [1–3]. Due to the advantages of abilities of artificial neural networks (ANNs) for nonlinear function approximation and high robust, the damage identification methodologies using ANNs have been widely researched over years.

Although there are many accepted damage identification methods using ANNs, the optimizations, such as the structures and parameters of ANNs, as well as the damage indexes, are researched and improved in order to obtain more accurate results. Vibration-based damage identification method that utilized ANNs to identify defects of an experimental model was proposed [4], where a steel beam was used as a structure and seven piezoelectric accelerometers were mounted on the top surface of the beams. With the beam response recorded from accelerometers and data acquisition system, the damage caused by cuts of the height was identified and the different damage cases were recognized as well. A seismic damage identification method based on artificial neural networks and modal variables was researched, which was verified with simulated data on a-5-storey office building [5]. However, it is found that the approach was quite sensitive to modal errors. Besides these acceleration and vibration based damage identification methods using ANNs were proposed, such as damage detection in a truss-type structure by means of vibration [6], damage identification in beam-like composite laminates by using the combination of natural frequencies and mode shapes as the input for ANNs [7], and so on. Furthermore, there were also strain-based damage identification methods that used ANNs, one of which is the prediction on crack positions and lengths of a lap-joint structure with the structural strain measurements as the input of ANNs [8].

However, the inputs of the ANNs are the features or measurements from single type of sensor, while suitable measurements from various types of sensors and data mining in various measurements can support more effective results [9]. Thus, this paper focuses on the inputs of ANNs; the multiscale inputs to ANNs are used to discuss the effectiveness of damage identification for large span structure.

The rest of the paper is organized as follows. In Section 2 the strategy to identify the damage by using artificial neural networks is firstly proposed, especially the construction on input parameters. In Section 3 a real large span space structure is introduced, including the dimensions, the characteristics, and the transient seismic analysis. In Section 4 the influences by using different parameters, inputs of ANNs, and different levels of noises are compared, while the conclusions are offered in the final section of the paper.

## 2. Damage Identification Using ANNs

### 2.1. The Structure of BP Neural Networks

The damage identification method is carried out by BP neural networks (BPNNs), whose full name is Back-Propagation Network. BPNN is a multilayer network, in which the weight value is trained by nonlinear differential equation. Because of the simple structure and fabricability of the BPNNs, it has been widely used in many research fields including function approximation, pattern recognition, information classification, data compression, and so on. However, there is still no clear criterion to determine the most appropriate network architecture for a certain system. Some scholars have proved that the neural network structure with two hidden layers can get better recognition results [10], so the BPNN with two hidden layers is taken into the consideration of the damage identification in this paper. Except for determining the input vector of the neural network-based damage identification, the optimization of the neural network structure also needs to consider several important parameters including the selection on the number of training sample sets, the number of neurons in hidden layers, the transfer function, and the training function.

*Transfer Functions and Training Functions*. The BPNNs for damage identification have one input layer, two hidden layers, and one output layer. The transfer functions are the tan-sigmoid function, the linear function, and linear function, respectively. There are many kinds of training functions, while different training functions are suitable for the different neural network structure [11]. The training functions used to discuss the optimal neural network in the paper are listed as (1) Levenberg-Marquardt algorithm [12], (2) Scaled Conjugate Gradient algorithm [13], (3) the Resilient Back-Propagation algorithm [14], (4) Gradient Descent with Momentum algorithm [15], (5) Gradient Descent with Momentum and Adaptive Learning Rate algorithm [15], (6) Fletcher-Reeves Conjugate Gradient algorithm [16], and (7) the BFGS Quasi-Newton algorithm [17]. The training functions are abbreviated in T-LM, T_SCG, T_RP, T_GDM, T_GDX, T_CGF, and T_BFG.

*The Number of the Neurons in Hidden Layers*. The optimal number of neurons in a hidden layer varies with different input and output, which is usually achieved by reiterative trials and accumulated experience [18]. The neuron number of the hidden layers may be approximately determined by the following equation [19]:

(1) $$\begin{array}{c} i=\sqrt{m+n}+A, \end{array}$$

where *i*, *m*, and *n* are the numbers of neurons in hidden layer, input layer, and output layer, respectively.  *A*  denotes an empirical constant, commonly ranging from 4 to 8 depending on the actual system.

### 2.2. The Inputs of Neural Network

The inputs of the neural network for damage identification are selected as three scenarios: one is the strain damage parameter, one is the acceleration damage parameter, and the last one is the multiscale damage parameter combined with strain damage parameter and acceleration damage parameter [20].

The strain damage parameter vector is defined as

(2) $$\begin{array}{c} d_{\varepsilon }=\left[\begin{array}{cccccc} d_{\varepsilon 1} & d_{\varepsilon 2} & \cdots & d_{\varepsilon i} & \cdots & d_{\varepsilon n} \end{array}\right], \end{array}$$

where *d*_*εi*_ is the normalized strain damage parameter vector generated by the strain time series measured from the *i*th  selected strain sensor.

The acceleration damage parameter vector is defined as

(3) $$\begin{array}{c} d_{\phi }=\left[\begin{array}{cccccc} d_{\phi 1} & d_{\phi 2} & \cdots & d_{\phi i} & \cdots & d_{\phi m} \end{array}\right], \end{array}$$

where *d*_*ϕi*_ is the damage parameter generated by the *i*th  selected mode shape.

The multiscale damage parameter vector is defined as

(4) $$\begin{array}{c} d_{\phi \varepsilon }=\left[\begin{array}{cc} d_{\phi } & d_{\varepsilon } \end{array}\right]=\left[\begin{array}{cccccccc} d_{\phi 1} & d_{\phi 2} & \cdots & d_{\phi m} & d_{\varepsilon 1} & d_{\varepsilon 2} & \cdots & d_{\varepsilon \text{n}} \end{array}\right]. \end{array}$$

### 2.3. The Outputs of Neural Network and Evaluation

For the same input vectors of the neural network, different network architectures take different identification results. In order to evaluate whether the architecture of neural network is the optimal one or not, the absolute average error $\overset{-}{e}$ is taken as the selection criterion. For a given input of neural network and network architecture, *m* times tests are carried out to test the neural network model. The absolute average error $\overset{-}{e}$ is defined as

(5) $$\begin{array}{c} \overset{-}{e}=\frac{\left(e_{1}+e_{2}+\cdots +e_{m}\right)}{m},\\ e_{i}=\left|\frac{\sum \alpha_{j}^{\prime }-\sum \alpha_{j}}{\sum \alpha_{j}}\right|\;\left(i=1,2,\ldots ,m;j=1,2,\ldots ,q\right), \end{array}$$

where *α*_*j*_′ and *α*_*j*_ are the identification value and theoretical value of damage extent on *j*th  damage location, respectively.

Because the noise is usually accompanied by the measurements from sensors, the noisy measurements are given in different noise levels. The robustness of the proposed method can be proofed by estimating the antinoise performance of damage identification method. Here the noise level is the ratio of the root mean square (RMS) of the noise to the RMS of the signal time series [21], which can be defined as

(6) $$\begin{array}{c} e=\frac{r_{n}}{r_{t}}\times 100\%, \end{array}$$

where *e* is the noise level; *r*_*n*_ is the root mean square of noise; and *r*_*t*_ is the root mean square of the signal time series.

## 3. Researched Structure

### 3.1. Structure Description and Its Finite Element Model

**Figure 1:**
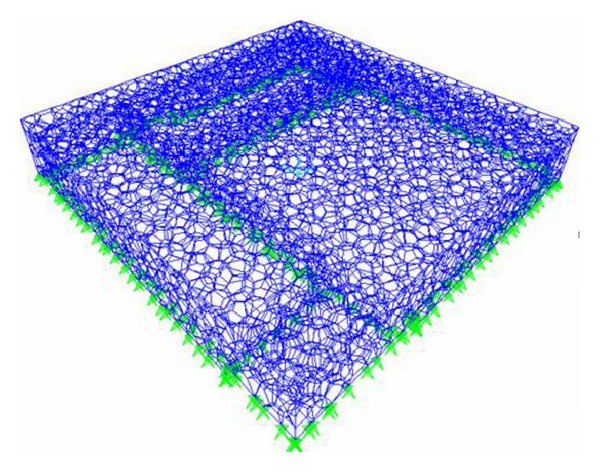
The finite element model of Beijing National Aquatics Center.

The steel superstructure of Beijing National Aquatics Center is a new kind of polyhedron spatial frame structure, whose outside size is 176.5389 m in length, 176.5389 m in width, and 29.3786 m in height. Polyhedron space has high repeatability, where the polyhedral cell in internal structure just needs four kinds of rod length and three types of nodes. The steel structure of National Aquatic Center is analyzed by finite element software SAP2000, and the node is set to be rigid connection and the member is set to be space beam element. The members are subjected to bending moment, shear, tension, or compression and torsion, simultaneously [22]. Its finite element model is shown in Figure 1.

### 3.2. Modal Analysis

The main natural frequencies of the intact structure can be obtained from structural modal analysis, which are shown in Table 1. The orders of the mode, the frequency values, and the mass participation factors to the corresponding mode are listed in Table 1, where the first six main modes are listed for each vibration direction.

### 3.3. Seismic Effect Analysis

**Figure 2:**
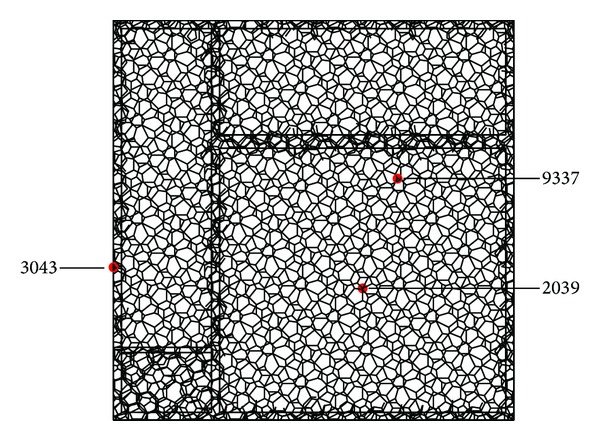
The displacement peak points.

**Figure 3:**
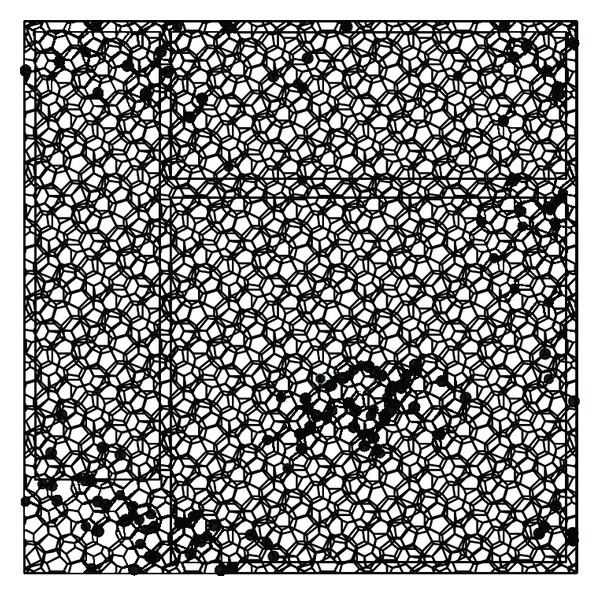
The plastic hinge state of roof by moment *M*_*y*_.

**Figure 4:**
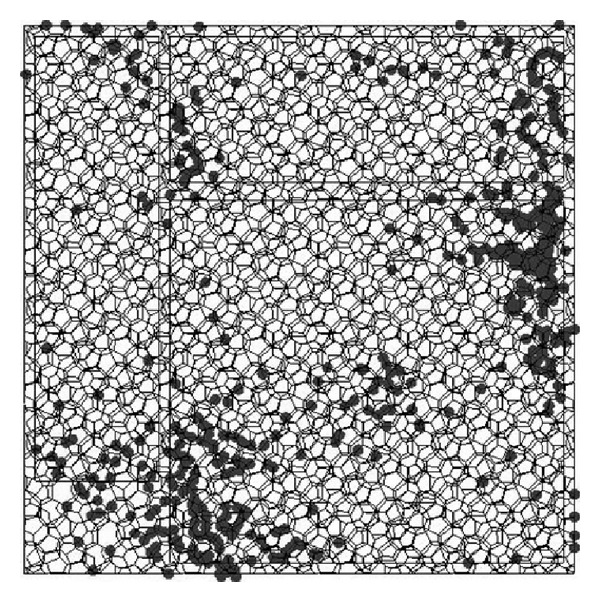
The plastic hinge state of roof by moment *M*_*z*_.

The structural analysis for the polyhedron space frame is that the members are subjected to the normal force and biaxial bending moments, while most joints are not subjected to lateral force and the lateral force is so small to a small number of members. The maximum moment occurs at the two ends of member and the bending moments on the two ends are almost in the opposite direction. The analysis results for the structure which is subjected to seismic action in three directions [22] are described as follows: (1) the displacement peak value in *Z* direction is 0.735 m, which occurs at the node (number 2039) located at the center of the long span roof (Figure 2); (2) the displacement peak value in *Y* direction is 0.322 m, which occurs at the node (number 9337) located at the end of one bottom chord of roof (Figure 2); (3) the displacement peak value in *X* direction is 0.286 m, which occurs at the node (number 3043) located at the surface of left wall (Figure 2); (4) the range of the normal force to the member is from −4.49017 × 10^3^ kN to 4.3187 × 10^3^ kN, while the top chord members are subjected to the maximum compression and the bottom chord members are subjected to maximum tension; and (5) the plastic hinge state of roof based on the dynamic elastoplastic analysis is shown in Figures 3 and 4 [23], where the structure is subjected to rare seismic action.

### 3.4. Damage Model and Load Cases

**Figure 5:**
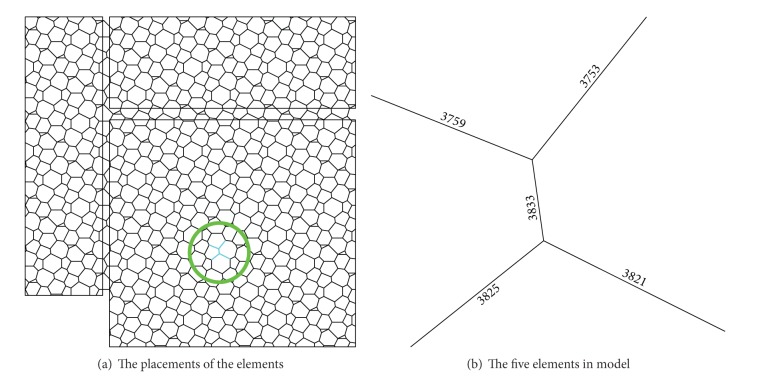
The placements of the five damaged elements.

The five bottom chord members around the node number 2039 are selected to be the damage case according to the analysis result of the structure which is subjected to seismic action. The selected damage locations are shown in Figure 5. In SAP2000, the structural damage extent is simulated by changing the elastic modulus of steel elements, so the different damage extents can be simulated by changing the elastic modulus of the five elements. The reduced elastic moduli of the five elements for simulating minimum and maximum damage extents are 0.95*E* and 0.50*E*, respectively. The 0.03*E* is taken to be the damage extent interval; the 16 damage extent scenarios and 16 structural damage models can be simulated finally.

**Figure 6:**
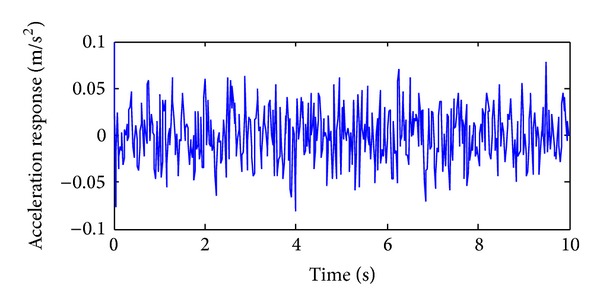
The acceleration time series in *X* direction of the first load case.

**Figure 7:**
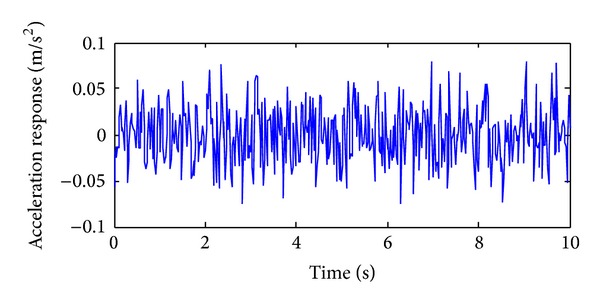
The acceleration time series in *Y* direction of the first load case.

**Figure 8:**
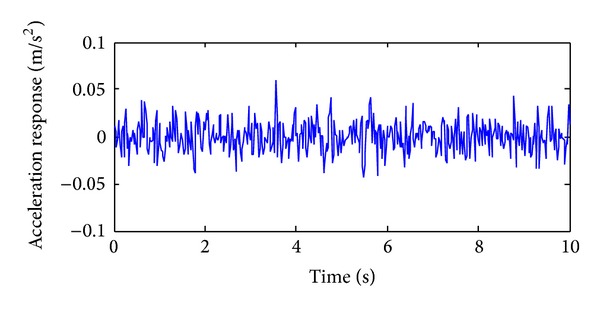
The acceleration time series in *Z* direction of the first load case.

The training, validation and prediction data for BPNN are from the structural analysis results on various damage models which are subjected to 36 kinds of earth pulsation load cases and the earth pulsations are simulated by white noises. For the first load case, the amplitudes of simulated white noise in *X*, *Y*, and *Z* direction are, respectively, 0.10, 0.08, and 0.06; that is, the acceleration amplitudes of simulated earth pulsations in these directions are 0.10 m/s^2^, 0.08 m/s^2,^ and 0.06 m/s^2^, respectively; the frequency range is from 0.5 Hz to 20 Hz; the period is 10 s and the time step is 0.02 s. The time series of simulated white noise in three directions are shown in Figures 6, 7, and 8.

For other load cases, amplitudes of each white noise in three directions only are changed, which implies that the acceleration amplitudes of earth pulsations in three directions are changed. The amplitudes of 36 kinds of simulated earth pulsations are shown in Table 2. So there are totally 576 kinds of damage cases data, as there are 16 kinds of damage models and 36 kinds of load cases. The data separation for training group, validation group, and prediction group are shown in Table 3.

According to Tables 3 and 6 scenarios are included in validation group; 6 scenarios are included in prediction group and 336 scenarios are included in the training group.

## 4. Results and Discussions

### 4.1. Sensitivity Analysis on Damage Parameter Index

#### 4.1.1. Sensitivity Analysis on Strain Damage Parameter and Its Selection

The MAC value is used to evaluate the sensitivity of the selected damage parameters, where the smaller value of MAC is, the more sensitivity of the damage parameter is [20].

**Figure 9:**
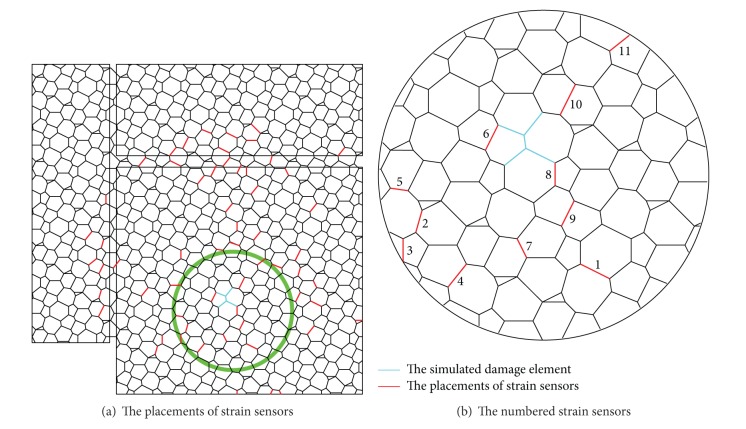
The previous placements of strain sensors.

The candidate placements for strain sensors are shown in Figure 9, which are labeled as *s*1, *s*2, *s*3, *s*4, *s*5, *s*6, *s*7, *s*8, *s*9, *s*10, and *s*11, respectively. By optimizing the placements of strain sensors and the selection of time steps, more sensitive strain damage parameter vectors can be determined.

Five scenarios on the number and placements of strain sensors are set as follows: (1) *s*1, *s*2, *s*3, *s*4, *s*5, *s*6, *s*7, *s*8, *s*9, *s*10, and *s*11; (2) *s*6, *s*7, *s*8, *s*9, *s*10, and *s*11; (3) *s*6, *s*7, *s*8, *s*9, and *s*10; (4) *s*6, *s*8, *s*9, and *s*10; and (5) *s*6, *s*8, *s*10, respectively. Six kinds of time step are defined to discuss the optimal strain damage parameter, which are five steps, seven steps, nine steps, eleven steps, thirteen steps, and fifteen steps, respectively.

For each strain damage parameter vector, the calculation results of sensitivity are shown in Table 4. The MAC values, compared between the damage scenario where the structure is in 0.95*E* damage and subjected to the 2nd load case and the damage scenario where the structure is in 0.50*E* damage and subjected to the 7th load case, are shown in Table 4. It can be seen from Table 4 that the minimum value of MAC is 0.005, where the strain damage parameters are from the 3rd scenario on the number and placement of strain sensors and the 15 time steps. Thus there are 75 elements in the strain damage parameter vectors, while the number of neurons for the input layer of BPNN is 75.

**Figure 10:**
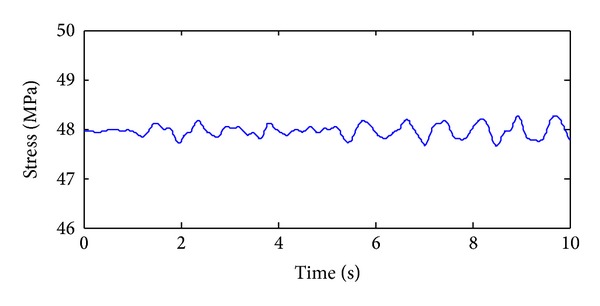
The time series of the strain sensor without noise.

**Figure 11:**
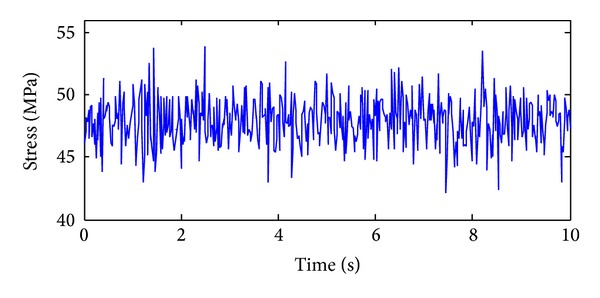
The time series of the strain sensor with noise.

**Figure 12:**
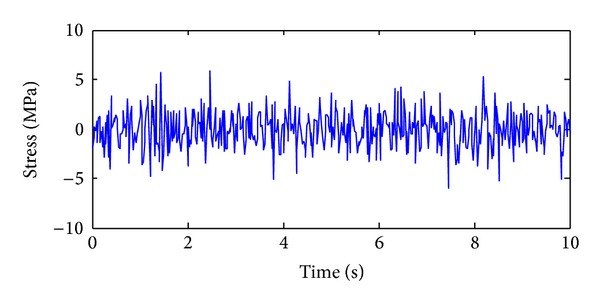
The time series of noise with 4% noise level.

In order to validate the effectiveness of the strain damage parameter, the noisy measurements are considered, where the strain measurements, the noisy strain measurements, and the noise are shown in Figures 10, 11, and 12, respectively.

The MAC values of strain damage parameter vector using noisy measurements are compared in Tables 5 and 6. The noise levels are 0.01, 0.02, 0.04, 0.08, and 0.10. The MAC values, compared between the damage scenario where the structure is in 0.95*E* damage and subjected to the 2nd load case and the damage scenario where the structure is in the corresponding damage extents in the table and subjected to the 7th load case, are shown in Table 5. The MAC values, compared between the damage scenario where the structure is in 0.95*E* damage and subjected to the 2nd load case and the damage scenario where the structure is in the corresponding damage extents in the table and subjected to the 2nd load case, are shown in Table 6.

It can be known from Tables 5 and 6 that the values of MAC are becoming smaller with the increase in damage extent when the measurements are without noises, which is in accordance with the theory analysis. In the opposite side, the variation on the value of MAC is becoming inconspicuous when the measurements are noisy.

#### 4.1.2. Sensitivity Analysis on Acceleration Damage Parameter and Its Selection

**Figure 13:**
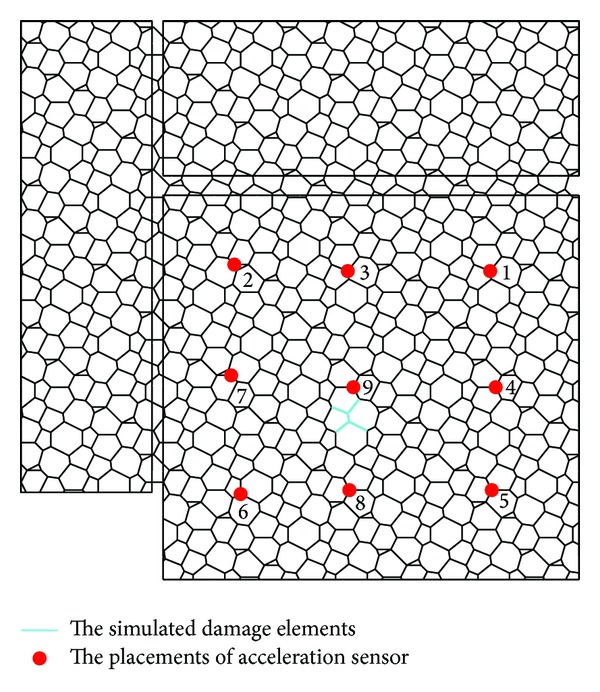
The previous placements of accelerometer.

The candidate placements for accelerometers are shown in Figure 13, which are labeled as *a*1, *a*2, *a*3, *a*4, *a*5, *a*6, *a*7, *a*8, and *a*9, respectively. By optimizing the placements of accelerometers and the selection of mode shapes, more sensitive acceleration damage parameter vector can be determined.

Five scenarios on the number and placements of accelerometers are set as follows: (1) *a*1, *a*2, *a*3, *a*4, *a*5, *a*6, *a*7, *a*8, and *a*9; (2) *a*2, *a*5, *a*6, *a*7, *a*8, and *a*9; (3) *a*4, *a*5, *a*6, *a*7, *a*8, and *a*9; (4) *a*3, *a*4, *a*7, *a*8, and *a*9; and (5) *a*6, *a*7, *a*8, and *a*9, respectively. Six modes are selected to discuss the optimal acceleration damage parameter in three vibration directions separately.

For each acceleration damage parameter vector, the calculation results of sensitivity are shown in Tables 7–9. The MAC values, compared between the damage scenario where the structure is in 0.95*E* damage and subjected to the 2nd load case and the damage scenario where the structure is in 0.50*E* damage and subjected to the 7th load case, are shown in Tables 7–9. It can be seen from Table 7 that the smaller values of MAC are from the 2nd, 7th, 10th, and 14th modes, while the number and placement of accelerometers are from the 1st scenario. It can be seen from Table 8 that the smaller values of MAC are from the 4th, 17th, and 19th modes, while the number and placement of accelerometers are from the 3rd scenario. It can be seen from Table 9 that the smaller values of MAC are from the 3rd, 16th, 27th, and 29th modes, while the number and placement of accelerometers are from the 1st scenario. Thus there are 90 elements in the acceleration damage parameter vectors, while the number of neurons for the input layer of BP neural network is 90.

**Figure 14:**
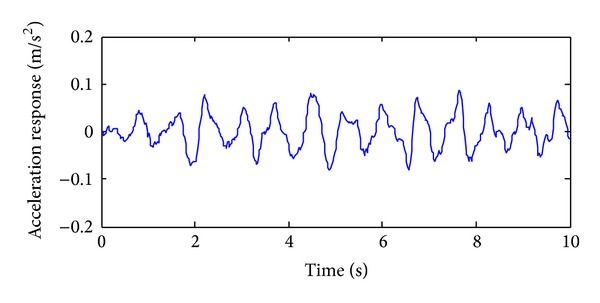
The time series of the accelerometer without noise.

**Figure 15:**
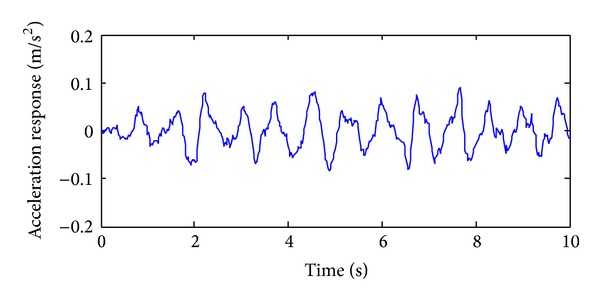
The time series of the accelerometer with noise.

**Figure 16:**
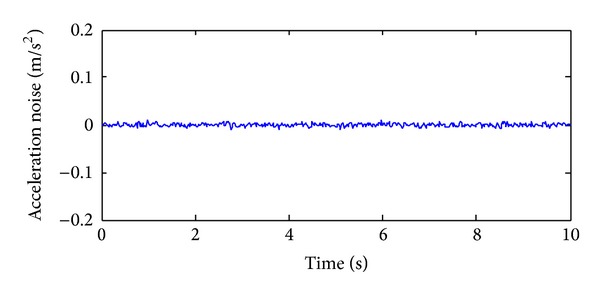
The acceleration noise with 10% noise level.

In order to analyze the effectiveness of the selected acceleration damage parameter vectors, the MAC values on noisy acceleration measurements are discussed. The acceleration measurements, the noisy acceleration measurements, and the noise are shown in Figures 14, 15, and 16, respectively.

The MAC values of acceleration damage parameter vector using noisy measurements are compared in Tables 10 and 11. The noise levels are 0.01, 0.02, 0.04, 0.08, and 0.10. The MAC values, compared between the damage scenario where the structure is in 0.95*E* damage and subjected to the 2nd load case and the damage scenario where the structure is in the corresponding damage extents in the table and subjected to the 7th load case, are shown in Table 10. The MAC values, compared between the damage scenario where the structure is in 0.95*E* damage and subjected to the 2nd load case and the damage scenario where the structure is in the corresponding damage extents in the table and subjected to the 2nd load case, are shown in Table 11.

It can be known from Tables 10 and 11 that the value of MAC is becoming smaller with the increasing in damage extent when the measurements are without noises, which is in accordance with the theory analysis. However, the variation on the value of MAC is becoming inconspicuous when the measurements are noisy.

#### 4.1.3. Sensitivity Analysis on Multiscale Damage Parameter

Based on the optimal selection on the strain damage parameter and the acceleration damage parameter, the sensitivity of multiscale damage parameter is analyzed. The MAC values of multiscale damage parameter vector using noisy measurements are compared in Tables 12 and 13. The noise levels are 0.01, 0.02, 0.04, 0.08, and 0.10. The MAC values, compared between the damage scenario where the structure is in 0.95*E* damage and subjected to the 2nd load case and the damage scenario where the structure is in the corresponding damage extents in the table and subjected to the 7th load case, are shown in Table 12. The MAC values, compared between the damage scenario where the structure is in 0.95*E* damage and subjected to the 2nd load case and the damage scenario where the structure is in the corresponding damage extents in the table and subjected to the 2nd load case, are shown in Table 13.

It can be seen from Tables 12 and 13 that the multiscale damage parameter vector is more sensitive to the damage extent compared with both the strain damage parameter vector and the acceleration damage parameter.

### 4.2. Selection on Architecture of Neural Networks

#### 4.2.1. The Number of the Training Groups

Five kinds of training groups are chosen to obtain the optimal predicted values which can be used to compare the effectiveness of the multiscale damage parameter and sole-scale damage parameter. Based on the training group and all the 24 kinds of load cases listed in Table 3, the front 8, 10, 11, 12, and 14 kinds of damage models are selected separately to give the different number of training groups, which are 192 training groups, 240 training groups, 264 training groups, 288 training groups, and 336 training groups.

#### 4.2.2. The Number of Neurons in Hidden Layers

According to the empirical equation (1) and by taking the strain damage parameter vector as the input and assuming that the constant value for the first hidden layer is A1 and the constant value for the second hidden layer is A2, there are four given choices on the number of neurons in hidden layers, which are shown in Table 14. For the acceleration damage parameter vector, the same number of neurons in hidden layers is selected to be discussed.

### 4.3. Damage Identification Based on Sole-Scale Measurements

The data from validation groups are used to choose the optimal architecture of the neural networks which have the different sets and number of neurons in the hidden layer. It is shown in Table 15 that the training function T-LM is used, while the optimal architecture of neural network is chosen by the minimum value of the maximum errors based on different noise level interferences. Similarly, the optimal architectures of neural networks using the different training functions are chosen and listed in Tables 16 and 17.

It can be known from Tables 16 and 17 that the training functions for the optimal architecture of neural networks with the strain damage parameter or acceleration damage parameter are both the Levenberg-Marquardt algorithm. The data from prediction groups are used to evaluate the effectiveness of the neural networks, where the optimal architecture of neural network is used. The prediction errors and maximum errors for each scenario in prediction group are shown in Tables 18 and 19. Meanwhile, the average errors are calculated based on all the prediction scenarios which are in the same noise level and they are shown in Table 20. It can be seen from Table 20 that the maximum average errors using the strain damage parameter and acceleration damage parameter are 16.1% and 16.5%, respectively, which are based on their own optimal neural network architecture.

### 4.4. Damage Identification Using Multiscale Measurements

The multiscale damage parameter, combined with the strain damage parameter and the acceleration damage parameter, is used to discuss the effectiveness of damage evaluation. The same procedure on selecting the optimal architecture of neural network is processed to the multiscale damage parameter, where the Levenberg-Marquardt algorithm is the best training function for multiscale damage parameter. The errors of the prediction scenarios are listed in Table 21 and the errors comparison of using different damage parameters with the optimal architectures of neural networks is shown in Table 22. It can be seen from Table 22 that the maximum error using multiscale damage parameters is obviously smaller than that using strain damage parameter or acceleration damage parameter, while the prediction results based on multiscale damage parameter are better than those based on strain damage parameter or acceleration damage parameter. Simultaneously the identification results show that the proposed damage identification method based on multiscale damage parameter can effectively synthesize the measured information from two kinds of sensors and provide better identification results.

## 5. Conclusions

The paper proposed an effective damage identification method based on the multiscale measurements from strain sensors and accelerometers using artificial neural networks. The conclusions are obtained as follows.

1. According to the principle of MAC, the method of sensitivity analysis on structural damage index is given and the optimal damage parameter vector is determined.
2. The optimal architectures of neural networks using the strain damage parameter and acceleration damage parameter as the input are discussed and selected, while the identification errors are listed for each scenario in prediction groups.
3. The optimal architecture of neural networks using the multiscale damage parameter as the input is discussed and selected, while the identification errors are compared with those using the sole-scale damage parameter, such as strain damage parameter and acceleration damage parameter. The proposed damage identification method is proofed to be effective and the identification errors of performance are better.
4. The proposed method can be used for a real large span space structure and the elastoplastic analysis should be processed at the beginning of selecting unfavorable structural members.

## Figures and Tables

**Table 1:** The main natural frequencies of the intact shell structure.

*X* transitional			*Y* transitional			*Z* transitional		
Mode	Period(s)	Mode mass participation factor	Mode	Period(s)	Mode mass participation factor	Mode	Period(s)	Mode mass participation factor
2	0.780	0.773	1	0.839	0.753	3	0.734	0.215
4	0.599	0.033	4	0.599	0.026	8	0.484	0.004
7	0.491	0.022	7	0.491	0.038	16	0.351	0.017
10	0.425	0.010	9	0.443	0.014	27	0.298	0.017
14	0.359	0.005	17	0.338	0.003	28	0.296	0.079
18	0.334	0.004	19	0.333	0.010	29	0.289	0.048

**Table 2:** The values of the load cases.

Number	*X* direction (m/s^2^)	*Y* direction (m/s^2^)	*Z* direction (m/s^2^)
1	0.100	0.080	0.060
2	0.150	0.120	0.090
3	0.175	0.140	0.105
4	0.200	0.160	0.120
5	0.225	0.180	0.135
6	0.250	0.200	0.150
7	0.275	0.220	0.165
8	0.300	0.240	0.180
9	0.325	0.260	0.195
10	0.350	0.280	0.210
11	0.375	0.300	0.225
12	0.400	0.320	0.240
13	0.425	0.340	0.255
14	0.450	0.360	0.270
15	0.475	0.380	0.285
16	0.500	0.400	0.300
17	0.525	0.420	0.315
18	0.550	0.440	0.330
19	0.575	0.460	0.345
20	0.600	0.480	0.360
21	0.625	0.500	0.375
22	0.650	0.520	0.390
23	0.675	0.540	0.405
24	0.700	0.560	0.420
25	0.725	0.580	0.435
26	0.750	0.600	0.450
27	0.775	0.620	0.465
28	0.800	0.640	0.480
29	0.825	0.660	0.495
30	0.850	0.680	0.510
31	0.875	0.700	0.525
32	0.900	0.720	0.540
33	0.925	0.740	0.555
34	0.950	0.760	0.570
35	0.975	0.780	0.585
36	1.000	0.800	0.600

**Table 3:** The data for training, validation, and prediction.

Group	Damage model (elastic modulus after damage)	Load cases (number, as shown in Table 2)
Validation group	0.80*E*	6, 12, 18, 24, 30, 36
Prediction group	0.65*E*	5, 10, 15, 20, 25, 31
Training group	The other 14 kinds	The other 24 kinds

**Table 4:** The MAC values of damage parameter induced by strain sensors.

Time steps	Scenario on the number and placements of strain sensors				
	1	2	3	4	5
5	0.921	0.598	0.803	0.610	0.216
7	0.727	0.362	0.669	0.290	0.372
9	0.403	0.203	0.002	0.048	0.338
11	0.903	0.679	0.844	0.715	0.342
13	0.631	0.441	0.761	0.361	0.558
15	0.526	0.284	0.005	0.082	0.257

**Table 5:** The MAC values of stain damage parameter vector extracted from different load cases.

Noise levels	Damage extents				
	0.92*E*	0.83*E*	0.71*E*	0.59*E*	0.50*E*
0.00	0.0051	0.0050	0.0048	0.0047	0.0046
0.01	0.0477	0.0709	0.3940	0.2398	0.1891
0.02	0.0579	0.0650	0.2540	0.0139	0.0313
0.04	0.0001	0.2316	0.1417	0.0008	0.0180
0.08	0.0001	0.0373	0.0012	0.2914	0.0024
0.10	0.3863	0.0268	0.0010	0.0036	0.0118

**Table 6:** The MAC values of stain damage parameter vector extracted from the same load case.

Noise levels	Damage extents				
	0.92*E*	0.83*E*	0.71*E*	0.59*E*	0.50*E*
0.00	1.0000	0.9998	0.9989	0.9961	0.9905
0.01	0.4538	0.5548	0.2523	0.6695	0.0366
0.02	0.0072	0.0011	0.0037	0.0129	0.0000
0.04	0.0042	0.0068	0.0235	0.0261	0.0015
0.08	0.1638	0.1018	0.0064	0.0010	0.0000
0.10	0.0692	0.0682	0.2063	0.0292	0.0657

**Table 7:** The MAC values of acceleration damage parameter in *X* transitional direction.

Mode	Number of accelerometers				
	9	6	6	5	4
2	0.042	0.367	0.602	0.533	0.049
4	0.055	0.522	0.033	0.533	0.906
7	0.055	0.264	0.092	0.335	0.611
10	0.036	0.216	0.440	0.954	0.224
14	0.001	0.010	0.002	0.331	0.001
18	0.242	0.405	0.348	0.472	0.148

**Table 8:** The MAC values of acceleration damage parameter in *Y* transitional direction.

Mode	Number of accelerometers				
	9	6	6	5	4
1	0.234	0.933	0.608	0.230	0.581
4	0.010	0.861	0.002	0.060	0.810
7	0.009	0.779	0.062	0.162	0.046
9	0.086	0.900	0.204	0.417	0.268
17	0.100	0.648	0.085	0.071	0.737
19	0.221	0.842	0.231	0.020	0.669

**Table 9:** The MAC values of acceleration damage parameter in *Z* transitional direction.

Mode	Number of accelerometers				
	9	6	6	5	4
3	0.003	0.027	0.102	0.423	0.007
8	0.397	0.021	0.222	0.464	0.044
16	0.081	0.299	0.024	0.201	0.004
27	0.039	0.041	0.236	0.014	0.082
28	0.039	0.041	0.236	0.014	0.082
29	0.010	0.018	0.054	0.074	0.023

**Table 10:** The MAC values of acceleration damage parameter vector extracted from different load cases.

Noise levels	Damage extents				
	0.92*E*	0.83*E*	0.71*E*	0.59*E*	0.50*E*
0.00	0.1057	0.0843	0.0673	0.0568	0.0512
0.01	0.1601	0.1471	0.1013	0.0769	0.0765
0.02	0.0957	0.0912	0.0140	0.0146	0.0914
0.04	0.0301	0.0229	0.1233	0.0617	0.0809
0.08	0.6297	0.4090	0.3884	0.5173	0.3854
0.10	0.0028	0.0039	0.0024	0.0015	0.0039

**Table 11:** The MAC values of acceleration damage parameter vector extracted from the same load case.

Noise levels	Damage extents				
	0.92*E*	0.83*E*	0.71*E*	0.59*E*	0.50*E*
0.00	0.9999	0.9976	0.9868	0.9573	0.9064
0.01	0.9994	0.9832	0.9772	0.9725	0.9987
0.02	0.9659	0.9552	0.9213	0.9900	0.7671
0.04	0.9574	0.9265	0.9902	0.9799	0.9832
0.08	0.7293	0.2113	0.8939	0.1842	0.5951
0.10	0.9585	0.8510	0.2923	0.9885	0.3672

**Table 12:** The MAC values of multiscale damage parameter vector extracted from different load cases.

Noise levels	Damage extents				
	0.92*E*	0.83*E*	0.71*E*	0.59*E*	0.50*E*
0.00	0.0378	0.0297	0.0233	0.0194	0.0173
0.01	0.0578	0.1018	0.0877	0.0385	0.0632
0.02	0.0871	0.0632	0.0141	0.0145	0.0149
0.04	0.0014	0.0373	0.0001	0.0011	0.0003
0.08	0.0029	0.0028	0.0011	0.1455	0.0090
0.10	0.0001	0.0001	0.0013	0.0010	0.0028

**Table 13:** The MAC values of multiscale damage parameter vector extracted from the same load case.

Noise levels	Damage extents				
	0.92*E*	0.83*E*	0.71*E*	0.59*E*	0.50*E*
0.00	0.9998	0.9966	0.9805	0.9342	0.8550
0.01	0.9991	0.9799	0.9534	0.9685	0.9976
0.02	0.7203	0.7306	0.8239	0.9694	0.6904
0.04	0.2148	0.2052	0.3055	0.3178	0.0898
0.08	0.0291	0.1810	0.8410	0.0998	0.5351
0.10	0.3614	0.7199	0.2158	0.8808	0.0038

**Table 14:** The number of neurons in hidden layers of neural networks.

*A*1	*A*2	1st hidden layer	2nd hidden layer
4	2	13	6
6	2	15	6
6	4	15	7
8	5	17	8

**Table 15:** The identification errors of neural networks based on strain damage parameters.

Training functions	Sets	Neurons	Noise levels						Maximum error
			0.00	0.01	0.02	0.04	0.08	0.10	
T_LM	192	(13,6)	0.057	0.195	0.264	0.180	0.156	0.164	0.264
T_LM	192	(15,6)	0.118	0.365	0.202	0.182	0.231	0.230	0.365
T_LM	192	(15,7)	0.064	0.256	0.185	0.172	0.714	0.117	0.714
T_LM	192	(17,8)	0.066	0.346	0.252	0.153	0.172	0.198	0.346
T_LM	240	(13,6)	0.040	0.202	0.151	0.186	0.161	0.162	0.202
T_LM	240	(15,6)	0.068	0.156	0.151	0.134	0.279	0.127	0.279
T_LM	240	(15,7)	0.046	0.182	0.135	0.925	0.172	0.150	0.925
T_LM	240	(17,8)	0.077	0.343	0.194	0.246	0.165	0.157	0.343
T_LM	264	(13,6)	0.086	0.400	0.182	0.509	0.228	0.164	0.509
T_LM	264	(15,6)	0.239	0.380	0.174	0.750	0.149	0.160	0.750
T_LM	264	(15,7)	0.082	0.164	0.176	0.151	0.197	0.192	0.197
T_LM	264	(17,8)	0.041	0.245	0.222	0.163	0.309	0.172	0.309
T_LM	288	(13,6)	0.044	0.136	0.390	0.145	0.112	0.165	0.390
T_LM	288	(15,6)	0.144	0.157	0.196	0.142	0.122	0.149	0.196
T_LM	288	(15,7)	0.067	0.242	0.224	0.178	0.404	0.257	0.404
T_LM	288	(17,8)	0.095	0.158	0.268	0.109	0.128	0.141	0.268
T_LM	336	(13,6)	0.082	0.111	0.125	0.103	0.255	0.114	0.255
T_LM	336	(15,6)	0.039	0.149	0.245	0.126	0.273	0.193	0.273
T_LM	336	(15,7)	0.044	0.169	0.130	0.081	0.128	0.484	0.484
T_LM	336	(17,8)	0.040	0.322	0.196	0.149	0.067	0.055	0.322

**Table 16:** The choice of the best neural network based on the strain damage parameters.

Training functions	Sets	Neurons	Noise levels						Maximum error
			0.00	0.01	0.02	0.04	0.08	0.10	
T_LM	288	(15,6)	0.144	0.157	0.196	0.142	0.122	0.149	0.196
T_SCG	192	(15,6)	0.286	0.275	0.177	0.266	0.199	0.206	0.286
T_RP	264	(15,7)	0.261	0.229	0.391	0.308	0.295	0.335	0.391
T_GDM	192	(15,6)	1.073	0.572	1.031	1.633	0.826	1.580	1.633
T_GDX	240	(17,8)	0.493	0.304	0.392	0.593	0.297	0.087	0.593
T_CGF	192	(13,6)	0.251	0.214	0.169	0.153	0.268	0.305	0.305
T_BFG	192	(15,6)	0.499	0.484	0.198	0.184	0.184	0.240	0.499

**Table 17:** The choice of the best neural network based on the acceleration damage parameters.

Training functions	Sets	Neurons	Noise levels						Maximum error
			0.00	0.01	0.02	0.04	0.08	0.10	
T_LM	264	(13,6)	0.083	0.153	0.158	0.148	0.167	0.168	0.168
T_SCG	192	(15,7)	0.196	0.486	0.429	0.307	0.365	0.223	0.486
T_RP	240	(15,6)	0.075	0.194	0.296	0.276	0.261	0.256	0.296
T_GDM	288	(15,6)	0.838	1.008	1.190	0.679	1.021	1.462	1.462
T_GDX	240	(15,6)	0.172	0.105	0.274	0.195	0.204	0.332	0.332
T_CGF	240	(15,7)	0.156	0.230	0.151	0.173	0.201	0.197	0.230
T_BFG	192	(15,7)	0.303	0.428	0.339	0.193	0.211	0.144	0.428

**Table 18:** The identification errors of forecasted cases based on strain damage parameters.

Number	Damage extents	Load cases	Noise levels						Maximum error
			0.00	0.01	0.02	0.04	0.08	0.10	
1	0.65*E*	5	0.186	0.086	0.247	0.124	0.145	0.129	0.247
2	0.65*E*	10	0.013	0.022	0.084	0.097	0.274	0.048	0.274
3	0.65*E*	15	0.046	0.087	0.037	0.002	0.037	0.065	0.087
4	0.65*E*	20	0.030	0.041	0.278	0.021	0.114	0.102	0.278
5	0.65*E*	25	0.036	0.144	0.140	0.042	0.103	0.109	0.140
6	0.65*E*	31	0.019	0.104	0.180	0.138	0.108	0.165	0.180

**Table 19:** The identification errors of forecasted cases based on acceleration damage parameters.

Number	Damage extents	Load cases	Noise levels						Maximum error
			0.00	0.01	0.02	0.04	0.08	0.10	
1	0.65*E*	5	0.147	0.012	0.209	0.145	0.118	0.210	0.210
2	0.65*E*	10	0.108	0.112	0.077	0.244	0.086	0.041	0.244
3	0.65*E*	15	0.002	0.065	0.159	0.007	0.054	0.232	0.232
4	0.65*E*	20	0.071	0.265	0.104	0.146	0.006	0.034	0.265
5	0.65*E*	25	0.018	0.251	0.068	0.078	0.101	0.013	0.251
6	0.65*E*	31	0.011	0.283	0.029	0.108	0.061	0.174	0.283

**Table 20:** The identification errors of the forecasted cases based on optimal neural networks.

Input vector	Sets	Neurons	Noise levels						Maximum error
			0.00	0.01	0.02	0.04	0.08	0.10	
Strain	288	(15,6)	0.055	0.081	0.161	0.071	0.130	0.103	0.161
Acceleration	264	(13,6)	0.059	0.165	0.108	0.121	0.071	0.117	0.165

**Table 21:** The identification errors of forecasted cases based on multiscale damage parameters.

Number	Damage extents	Load cases	Noise levels						Maximum error
			0.00	0.01	0.02	0.04	0.08	0.10	
1	0.65*E*	5	0.179	0.100	0.009	0.043	0.021	0.003	0.179
2	0.65*E*	10	0.109	0.110	0.051	0.112	0.083	0.110	0.110
3	0.65*E*	15	0.173	0.152	0.118	0.135	0.006	0.122	0.173
4	0.65*E*	20	0.166	0.028	0.353	0.019	0.164	0.025	0.353
5	0.65*E*	25	0.065	0.005	0.061	0.109	0.218	0.053	0.218
6	0.65*E*	31	0.001	0.125	0.115	0.210	0.063	0.124	0.210

**Table 22:** The identification errors of forecasted cases based on three different input vectors.

Input vectors	Sets	Neurons	Noise levels						Maximum error
			0.00	0.01	0.02	0.04	0.08	0.10	
Strain	288	(15,6)	0.055	0.081	0.161	0.071	0.130	0.103	0.161
Acceleration	264	(13,6)	0.059	0.165	0.108	0.121	0.071	0.117	0.165
Combination	336	(19,6)	0.059	0.096	0.118	0.126	0.085	0.106	0.126
